# Private Sector Hospitals’ Response to Changes in Demand for Health Insurance in Arab Countries

**DOI:** 10.36469/001c.140416

**Published:** 2025-06-20

**Authors:** Vivian Nasiruddin

**Affiliations:** 1 Department of Economics King Abdulaziz University, Jeddah, Saudi Arabia

**Keywords:** private sector hospitals, demand, health insurance

## Abstract

**Background:** Health insurance (HI) plays a vital role in providing health services, as it covers relevant healthcare costs to improve health outcomes. **Objectives:** The study aimed to analyze the extent to which private sector hospitals respond to changes in demand for HI in 3 Arab countries between 2006 and 2022. Methods: A structural equation model was used to evaluate the dynamic association between the variables. The study sample comprised the Kingdom of Saudi Arabia, the United Arab Emirates, and Jordan. **Results:** There were largely negative relationships between the demand for insurance and indicators of healthcare capacity, namely, the number of hospitals, hospital beds, and nurses. This suggests that publicly funded and organized healthcare systems limit private insurance demand. Furthermore, the panel vector autoregression Granger causality tests indicated a dynamic, 2-way Granger causal relationship between insurance demand and infrastructure. The policy implications of the study therefore suggest recommendations that healthcare planning be coordinated with insurance policy planning. **Discussion:** Regarding the number of hospitals, the coefficient for demand for HI was -0.169 (*P* = .032, indicating a negative but not significant relationship between HI and the number of hospitals. For beds, the coefficient for HI was 0.0000574 (*P* <  .001), suggesting a statistically significant negative relationship between HI and number of beds. Regarding the number of doctors, the coefficient for HI was -0.0000266 (*P*< .001), indicating a statistically significant negative relationship between HI and number of doctors. For the number of nurses, the coefficient for future insurance demand was -0.0000968 (*P* <  .001), reinforcing a negative relationship between HI and number of nurses. **Conclusions:** The study presents important insights into the intricate interplay between healthcare infrastructure and insurance demand in the private hospital markets of Saudi Arabia, the United Arab Emirates, and Jordan. These findings highlight the need for comprehensive health system planning in conjuction with insurance reforms, infrastructure development, and workforce reinforcement to maximize the sustainability and effectiveness of HI plans in the region.

## INTRODUCTION

The key driver of many national economies is the insurance sector, given the volume of its investments and the premiums it generates, as well as the social and economic role it plays through its coverage of various risks. Because it supports different markets and transactions, countries have adopted it as a primary engine for driving development and building a productive economy that does not depend on a single source.^1^ There are multiple types of insurance, which can be classified by the risk insured against, the practical management of the insuring body, freedom in insurance, and the insurance entity. Health insurance (HI), which depends on the insured risk, aims to protect the insured from the risk of injury and illness, with the insurer bearing some or all of the necessary medical care costs on behalf of the insured, in exchange for the insurance premium.^2^

While health care is considered a fundamental right,^3^ HI is essential for providing health services to all insured members of society, increasing utilization of both inpatient and outpatient care.^4^ As more people are insured, overall productivity and economic growth improve due to improved health status and disease prevention. HI also provides financial security by covering healthcare expenses,^5,6^ reducing the burden on individuals and lowers government healthcare expenditures.^7^

The demand for HI is affected by many factors (eg, social, economic, financial, health), the understanding and analysis of which can improve healthcare services and the overall health system. Several studies have found that higher income levels can positively impact INS,^8–10^ which in turn may stimulate investment in the healthcare infrastructure. Investment in the healthcare sector represents an increase in the volume of fixed assets such as land, buildings, and medical devices in the national economy, and thus increases national wealth.^11^ However, increased INS can also affect hospitals, which rely on HI payments.^12^ This requires hospitals to quickly respond, especially in resource-constrained environments like developing countries.^12^ It also requires the proper selection of these resources; for example, it has been observed in Germany that health ministers with a medical degree increase hospital capacity and financing through HI.^13^

Although HI reduces healthcare costs for individuals, it can increase demand for services, thereby putting pressure on hospitals to meet their demands.^14^ For example, hospitals may need to increase their capacity and infrastructure, such as increasing the number of beds, doctors, nurses, and hospitals themselves. In addition to providing adequate healthcare, increasing insurance coverage to achieve near-universal HI may lead to increased pressure on available medical resources. This raises many challenges regarding the quality and type of health services provided, in addition to the challenges in the intensity of treatment and healthcare experiences for patients, depending on the health, economic, and social status of the country.^11^

### Study Objectives

Accordingly, this study aims to analyze the extent to which private-sector hospitals respond to changes in demand for HI in some Arab countries between 2006 and 2022. To identify the general trend of the variables and their relationship, descriptive tests were conducted using the Stata statistical program. The HI sector stimulates economic growth; diversification, together with a healthy level of human capital, is important for achieving this growth.^15^ Therefore, alongside studying the role of HI in improving healthcare, it is equally important to examine the readiness to provide these services. From this standpoint, the research question can be formulated as follows: To what extent do private sector hospitals respond to changes in demand for HI?

The study aims to analyze the extent to which private sector hospitals respond to the change in demand for HI in the Kingdom of Saudi Arabia (KSA), the United Arab Emirates (UAE), and the State of Jordan between 2006 and 2022.

### Literature Review and Hypothesis Development

HI is a broad concept, depending on the ideological lens used. Many studies have found a positive impact of HI, such as maintaining the health of the individual and improving access to medical care, in addition to reducing the burden of costs of healthcare costs.^15^ For example, Al-Hanawi et al^16^ found that, despite the differences in demand for health services based on HI type, HI positively affects the conduct of medical examinations and periodic visits. Similarly, Memon et al^17^ analyzed the impact of the mother’s private HI on the weight of the fetus at birth in KSA during 2020-2021. The study discovered that increasing the period of maternal HI coverage caused a 95% reduction in the risk of low birth weight.

On the other hand, some studies have indicated the need to be cautious of HI policies to provide appropriate health coverage while controlling their cost rates. Trish and Herring^18^ found that HI premiums rise in markets with a high concentration of insurance companies, while those premiums decrease in markets with a high concentration of hospitals. Conversely, Dauda^19^ postulated that increasing concentration of the insurance company market leads to lower insurance premium prices, while increasing hospital market concentration leads to a price increase. Likewise, Dugan^20^ assessed the relationship between HI and patients’ demand for health services in the United States. The study revealed high rates of demand for preventive and routine care services, especially physician services, among insured people, thus highlighting the necessity of adopting awareness programs for equitable use of medical services.

While the demand for HI is affected by multiple factors that vary according to country and group setting, the levels of demand for HI vary, too. According to Owusu-Sekyere and Chiaraah,^8^ the factors influencing the demand for HI in Ghana were the family’s social and economic characteristics, financial situation, and health history. The study findings align with the results of Dror et al,^9^ which highlighted the same factors in rural India from 2010 to 2012. Additionally, Hillebrecht et al^21^ suggested following targeted strategies and designing insurance products that are aligned with the budgets of poor families to improve the HI system. Doiron and Kettlewell^10^ noted that young women in Australia who want to have children were more eager to buy HI.

Hospitals affect HI in terms of service, quality, and efficacy. Sepehri et al^22^ measured the extent to which hospital admissions and length of stay are affected by HI programs in Vietnam from 2001 to 2002. They reported an increase in the length of hospital stay for individuals insured by the compulsory HI programs and programs for the poor, thereby requiring more resources in hospitals to provide high-quality health care. However, the length of stay was not affected by the voluntary HI program. Correspondingly, Audibert et al^23^ investigated the efficiency of hospitals in China and the factors affecting them from 2000 to 2008. The study showed a decline in the technical efficiency of the hospitals over time, demonstrating their inability to use the available resources with optimal efficiency in providing health services and indicating that HI may not have a significant impact on improving hospital efficiency. Moreover, Abuosi et al^24^ observed that insured patients in Ghana had the advantage of easy access to good health services and that the overall quality of care was moderately satisfactory for all patients, whether insured or uninsured. Wang et al^25^ explained that the socioeconomic status of patients was positively associated with the choice of hospital rather than the social HI. Mukwena and Manyisa^26^ explored the need to prepare hospital to implement national HI by increasing human resources training, infrastructure, and medicines.

With the above in mind, this study formulated the following hypotheses:

H_1_: There is a significant direct relationship between the demand for HI and the number of hospitals (HPS).

H_2_: There is a significant direct relationship between the demand for HI and the number of beds (BED).

H_3_: There is a significant direct relationship between the demand for HI and the number of doctors (DCR).

H_4_: There is a significant direct relationship between the demand for HI and the number of nurses (NRS).

### Study Importance

The subject of the study derives its importance from the significance of the HI sector, the growth of which is supposed to contribute to improving the health services provided and thus the health status of society, supporting the entire national economy. Accordingly, determining the extent to which private sector hospitals can respond to changes in demand for HI should help in strategic planning for hospitals in the future. This, in turn, will help to identify the areas needing development while meeting the changes in demand for enhancing hospital efficiency. Therefore, this study can provide insights that may help in making sound decisions about planning processes and resource management, competitiveness and innovation, transparency and communication, patient safety, and ultimately increased productivity rates in society.

## METHODS

### Data and Variables

This study used demand for HI (proxied by gross written premiums) as the independent variable and a set of dependent variables (number of hospitals, number of beds, number of doctors, number of nurses) to reflect performance and responsiveness of private sector hospitals. In addition, control variables such as gross domestic product per capita (GDPPC) and population growth (POP) were also incorporated in the analysis to identify the appropriate influence on the dependent variable. On the basis of existing literature and the author’s conceptual framework, the dependent variables were selected. In addition, to handle the missing observations, this study employed the mean imputation method. The selection of countries and time frame was based on geographic region and availability of consistent data for all variables in the selected countries. Data for gross written premiums, HPS, BED, DCR, and NRS were retrieved from the official Ministry of Health websites of each respective country.^27–29^ World Bank websites were used to obtain the data for control variables. Further, to ensure comparability across KSA, UAE, and Jordan, all variables were harmonized. Gross written premiums were converted to constant 2020 USD using Consumer Price Index data. The variables and their definitions are presented in the **Appendix**.

### Model Specification

This study investigates the complex interrelationship among the examined variables through regression analysis. To control for heteroskedasticity and statistical dispersion, 17-year panel data for the 3 countries were utilized. To address reverse causality and the resulting endogeneity, we adopted Fornell and Larcker’s proposed simultaneous equation model (SEM),^30^ which integrates covariance structure analysis, factor analysis, multiple regression, and path modeling.^31^ This approach consists of 2 analytical models: (1) the measurement model used to evaluate latent variables, and (2) the structural model to measure bidirectional causality among endogenous variables.^32^ To conduct SEM analysis, 3 regression equations were formulated, corresponding to the HPS model, BED model, DCR model, and NRS model (**Equations 1-4**). SEM facilitates a systematic investigation of the dynamic interactions among the mentioned variables.

(1)HPSit=α0+α1Hit+α2 GDPPC it+α3 POP it+εit

(2)BEDit=β0+β1HIit+β2GDPPCit+β3POPit+ϵit

(3)DCRit=γ0+γ1HIit+γ2GDPPCit+γ3POPit+ϑit

(4)NRSit=δ0+δ1HIit+δGDPPCit+δ3POPit+ωit

The variables HPS, BED, DCR, and NRS denote the number of hospitals, beds, doctors, and nurses, respectively. HI, GDPPC, and POP imply the demand for HI, GDPPC, and POP, respectively. The subscript *it* represents the cross-section unit (country) *i* in the year *t*.

The SEM framework used consists of 3 exogenous variables and 4 observed endogenous variables. To analyze the estimation procedure of the SEM framework, Stata version 18 was used. In addition, model fit was evaluated using key parameters such as normed chi-square (χ²/*df*), Tucker-Lewis index, comparative fit index, coefficient of determination, standardized root mean squared, and root mean square error of approximation, in accordance with previous studies recomendations.^33^

Whereas the SEM model integrates causal factors, unit root testing is required to determine whether a variable is stationary. When all variables exhibit stationarity, a cointegration test is applied to verify the long-term relationships.

### Cross-sectional Dependence

Before analyzing the data, it is important to explore the features of the data sets. Using non-stationary data can result in spurious models and can produce misleading findings. To avoid the spurious, inconsistent, and biased panel data analysis, cross-dependence must be evaluated. The cross-dependence te/fst, introduced by Pesaran,^34^ is used to assess whether cross-sections are linked on a global or regional scale. **Equation 5** corresponds with the cross-dependence test:

(5)CD=2TN(N−1)(∑i=1N−1∑j=1+1NTijhij2)

where *N* indicates the size of cross-sections, *h* represents the correlation coefficient, and *T* implies the period. To test cross-dependence, the null and alternative hypotheses are stated as follows:

Null: No cross-sectional dependence

Alternative: Cross-sectional dependence present

### Panel Unit Root Test

In addition, the second-generation panel unit root test is used to effectively manage the issue of cross-dependence in panel data. This study adopted the framework developed by Pesaran and Yamagata,^35^ referred to as cross-sectionally augmented Im-Pesaran-Shin (CIPS) and cross-sectionally augmented Dickey-Fuller (CADF), for stationary tests. In the presence of heterogeneity and cross-dependence among the cross-sections, the CIPS test, shown in **Equation 6**, provides reliable outcomes:

(6)Δxit=αit+βixit−1+∂iT+∑j=1NθitΔxi,t−j+ϑit

where ∆ denotes the first-difference operator, *x^it^* indicates the examined variable across the cross-section unit and period, *x^it−1^* represents the lagged first differences used to address serial correlation across the error terms, refers to the intercept, and *T* and indicate time and error terms, respectively.

### Cointegration

Accordingly, **Equations 5 and 6** are formulated to conduct the cointegration test involving at least 2 variables. If variables are integrated of order *d*, denoted as *I(d),* they have a relationship. This study employed the Westerlund^36^ and Kao^37^ cointegration tests. Westerlund’s test^36^ improves panel cointegration by addressing cross-sectional dependence and heterogeneity, common in panel data. This structural approach, unlike residual-based methods, offers more robust results and is easy to implement. Extensive simulations show that the test has good size and power properties, making the empirical research valauble with complex panel data sets.

Kao,^37^ on the other hand, focuses on residual-based tests for cointegration in panel data and addresses the problem of spurious regression, which can occur when nonstationary panel data are used without proper adjustments. This

approach expands the Engle-Granger 2-step methodology to panel data, providing a framework to test for cointegration by examining the stationarity of residuals from panel regressions. Kao’s tests are designed to be straightforward and computationally efficient, making them accessible for applied work. They are particularly useful when the primary interest lies in understanding the long-term equilibrium relationships among variables in panel data sets.

Together, Westerlund^36^ and Kao^37^ have contributed to advance the methodology for testing cointegration in panel data. While Westerlund’s methods provide robust tools to handle structural dynamics and cross-sectional dependencies, Kao’s tests offer a simpler, residual-based approach for identifying long-run relationships. Both methodologies have their advantages and are complementary, providing researchers with a comprehensive toolkit for panel cointegration analysis. The advancements made by these scholars have facilitated more accurate and reliable empirical research in economics and other fields where panel data are prevalent.

### Causality-Based Vector Autoregression

To analyze the dynamic relationship and causality-based vector autoregression (VAR) between the insurance demand (INS), HPS, BED, DCR, and NRS, this research employed a panel VAR model, estimated using the generalized method of moments approach. Specifically, the study utilized an extended version of the Sims^38^ methodology, as adapted by Abrigo and Love.^39^ Although exogenous variables can be introduced into the panel VAR framework, it is more common to treat variables as endogenous within this context. This is particularly important when examining the shadow economy’s interactions with other economic factors, given its complex nature. The shadow economy refers to unreported or informal economic activity that is not subject to government regulation or taxation. It not only influences but is also influenced by various economic variables. In a VAR model, each variable is regressed on its own lagged values as well as the lagged values of other variables in the system. This allows for the simultaneous estimation of the dynamic interactions between the variables.^40^

In causality-based VAR, researchers aim to recognize the direction and strength of causal relationships between variables. This involves assessing whether changes in one variable cause changes in another variable and the extent to which these causal relationships exist. Causality in VAR models is typically assessed through various statistical tests, such as Granger causality tests, which examine whether past values of one variable provide additional information for predicting another variable beyond its past values.^41^

## RESULTS

### Cross-Dependence Test

The Pesaran cross-dependence^42^ and Fress^43^ tests were applied to evaluate the cross-dependence among the variables, before conducting the panel unit root test. Trend dependence among the variables, before conducting a panel unit root test. The features of data sets in **Table 1** demonstrates that both cross-dependence tests have significant values, indicating the rejection of (no cross-dependence). The results show *P* < .05, suggesting that the model is cross-sectionally dependent.

**Table 1.** Cross-Dependence Test

**Variables**	**Pesaran CD**	**Frees**
HI	8174.9b	121.7b
HPS	6520.4b	176.9c
BED	4369.2c	83.2c
DCR	2996.0b	131.5c
NRS	3278.5c	34.88b
GDPPC	5287.1a	66.3b
POP	8823.6c	43.5b

Abbreviations: CD, cross-dependence; BED, number of beds, DCR, number of doctors; GDPPC, gross domestic product per capita; HI, health insurance; HPS, number of hospitals; NRS, number of nurses; POP, population growth.

^a^*P* < .1.

^b^*P* < .05.

^c^*P* < .01.

### Panel Unit Root Tests

To generate unbiased and reliable results despite the appearance of cross-dependence in data, we adopted the second-generation panel unit root test. Therefore, CIPS and CADF tests are used to examine the stationarity of the examined variables. **Table 2** presents the results of the CIPS and CADF unit root test. It is observed that all variables HI, HPS, BED, DCR, NRS, GDPPC, and POP are stationary at the level. This indicates that all variables can be used in level form in subsequent analyses.

**Table 2.** Panel Unit Root Test

		**CADF**		**CIPS**	**Outcome**
	**I(0)**	**I(1)**	**I(0)**	**I(1)**	
HI	-2.340b	–	-2.47	–	I(0)
HPS	-2.476a	–	-2.553	–	I(0)
BED	-2.778a	–	-2.467	–	I(0)
DCR	-2.063a	–	-2.614	–	I(0)
NRS	-2.610b	–	-2.591	–	I(0)
GDPPC	-2.710a	–	-3.007	–	I(0)
POP	-2.346a	–	-2.772	–	I(0)

Abbreviations: BED, number of beds; CADF, cross-sectionally augmented Dickey-Fuller; CIPS, cross-sectionally augmented Im-Pesaran-Shin; DCR, number of doctors; GDPPC, gross domestic product per capita; HI, health insurance; HPS, number of hospital beds; NRS, number of nurses; POP, population growth.

Note: CIPS critical values are -2.21, -2.34, -2.6 at 10%, 5%, and 1%, respectively.

^a^*P* < .01.

^b^*P* < .05.

### Cointegration Tests

**Table 3** presents the results of various cointegration tests, including the Westerlund and Kao tests. The Westerlund cointegration test yields a statistic of 2.4863 (*P* = .040), indicating strong evidence of cointegration. The Kao cointegration test results are not provided. The modified Dickey-Fuller *t*-test of Kao cointegration shows a statistic of -2.6466 with a (*P* = .026), and the Dickey-Fuller *t*-test indicates a statistic of -2.891 (*P* = .003), both suggesting rejection of the null hypothesis of no cointegration at conventional significance levels. Results for the augmented Dickey-Fuller test are missing. The unadjusted versions of the modified Dickey-Fuller *t*-test and the Dickey-Fuller *t*-test show similar outcomes, with statistics of -2.658 (*P* = .0049) and -2.774 (*P* = .013), respectively, further supporting the presence of cointegration.

**Table 3.** Cointegration Tests

**Test**	**Statistic**	***P* Value**
Westerlund cointegration	2.4863	.040
Kao cointegration		
Modified Dickey-Fuller *t*	-2.6466	.026
Dickey-Fuller *t*	-2.891	.003
Augmented Dickey-Fuller *t*	–	–
Unadjusted modified Dickey-Fuller	-2.658	.0049
Unadjusted Dickey-Fuller *t*	-2.774	.013

These findings collectively provide robust evidence of cointegration among the variables, underscoring the long-term equilibrium relationship within the data set.

### SEM Analysis

The SEM findings are presented in **Table 4**. Based on the HPS equation, it is observed that a significant negative relationship is present between demand for HI and the number of private hospitals (*P* = .032), supporting *H_1_*. This indicates that a 1-unit increase in the demand for HI causes a decrease of 0.169 units in number of private hospitals. In addition, GDP per capita has a negatively significant effect on private hospitals (*P* < .001), where a 1-unit increase in GDP per capita decreases the number of private hospitals by 0.001 units.

**Table 4.** Simultaneous Equation Model Analysis

	**Coefficient**	**Robust SE**	***z* Value**	*****P > z*****	**95% CI**	
					**Upper**	**Lower**
HPS						
HI	-0.169	0.091	-1.860	.032	-0.347	0.009
GDPPC	-0.001	0.0003	-3.83	<.001	-0.002	-0.0007
POP	-3.877	1.042	-3.72	<.001	-5.920	-1.834
Constant	171.704	12.612	13.61	<.001	146.9843	196.424
BED						
HI	-.0000574	8.14e-06	-7.05	<.001	-0.000073	-0.000041
GDPPC	-0.257	0.056	-4.53	<.001	-0.369	-0.146
POP	-522.480	166.588	-3.14	.002	-848.987	-195.973
Constant	20078.18	1920.627	10.45	<.001	16313.82	23842.54
DCR						
HI	-0.0000266	0.00001	-1.44	.149	-0.000062	9.57e-06
GDPPC	-0.159	0.116	-1.37	.171	-0.388	0.068
POP	1612.831	351.188	-4.59	<.001	-2301.147	-924.5149
Constant	32571.12	4016.423	8.11	<.001	24699.08	40443.16
NRS						
HI	-0.0000968	0.0000231	-4.20	<.001	-0.000142	-0.0000516
GDPPC	-.0425059	.1879595	-0.23	.821	-.4108998	.325888
POP	-2569.447	526.8076	-4.88	<.001	-3601.971	-1536.923
Constant	40384.38	5499.449	7.34	<.001	29605.66	51163.1

Abbreviations: BED, number of beds; CADF, cross-sectionally augmented Dickey-Fuller; CI, confidence interval; CIPS, cross-sectionally augmented Im-Pesaran-Shin; DCR, number of doctors; GDPPC, gross domestic product per capita; HI, health insurance; HPS, number of hospital beds; NRS, number of nurses; POP, population growth.

SEM analysis based on the BEDS equation also supports *H*_2_, which asserts that HI is significantly associated with the number of beds in a private hospital. However, a negative relationship was found between them (*P* < .001), highlighting that 1-unit increases in HI result in decreases of 0.0000574 units in private hospital beds. Furthermore, it was found that GDPPC (*P* < .001) and POP (*P* = .002) are also negatively associated with a number of private hospital beds. Specifically, 1-unit increases in GDPPC and POP result in decreases of 0.257 and 522.480 units in hospital beds, respectively.

Regarding the number of doctors, HI (*P* = .149) and GDPPC (*P* = .171) show negative but statistically insignificant relationships, rejecting *H*_3_, which asserts that HI and DCR are significantly related. This finding suggests that insurance demand may not directly drive medical workforce growth, suggesting that a 1 unit increase in HI leads to a decrease of -0.0000266 number of doctors. However, population growth is found to have a significant negative impact (*P* < .001), with a 1-unit increase in POP leading to a decrease of 1612.831 doctors.

Lastly, the number of nurses is significantly and negatively influenced by both HI (*P* < .001) and POP (*P* < .001), where each unit increase in HI and POP reduces the number of nurses by 0.0000968 and 2569.447 units, respectively. These results support H_4_, which states the presence of a significant association between HI and NRS. Conversely, the GDPPC has a negative effect on nurses and is not statistically significant (*P* = .821), suggesting that broader economic indicators may not directly influence the nursing workforce unless mediated by policy or institutional factors.

**Table 5** presents the model fit indices, such as χ/*df*, root mean square error of approximation, Tucker-Lewis index, comparative fit index, standardized root mean squared, and coefficient of determination, complying with the guidelines presented by Hair et al. **Table 5** shows the model to be a good fit as it meets the recommended values.

**Table 5.** Model Fit Summary

**Fit Statistic**	**Recommended Values**	**Value**
χ^2^/*df*	<5	450.160
Root mean square error of approximation	<0.10	0.092
Tucker-Lewis index	>0.90	0.956
Comparative fit index	>0.90	1.0
Standardized root mean squared	<0.080 for a good fit, but 0.80-0.10 acceptable	0.080
Coefficient of determination	–	0.861

Abbreviation: *df*, degrees of freedom.

### Granger Causality: Robustness Check

The results of a panel VAR-Granger causality Wald test, specifically examining the relationship between the INS variable and all other variables in the model are shown in **Table 6**. The results revealed a significant relationship between the lead variable INS and the lag variables HPS, BED, DCR, and NRS (χ² = 17.099, *P* < .001).

**Table 6.** Panel VAR-Granger Causality Wald Test

**Lead Variable: INS**	**χ2**	***P* Value**
Lag variables: HPS, BED, DCR, and NRS	17.099	<.001

Abbreviations: BED, number of beds; CADF, cross-sectionally augmented Dickey-Fuller; CIPS, cross-sectionally augmented Im-Pesaran-Shin; DCR, number of doctors; GDPPC, gross domestic product per capita; HI, health insurance; HPS, number of hospital beds; NRS, number of nurses; POP, population growth.

The chi-square statistic for the test is 17.099 (*P* < .001). This *P* value is well below the conventional significance levels of 0.05, 0.01, and even 0.001, indicating strong evidence to reject the null hypothesis of no Granger causality. Therefore, the results suggest that INS Granger-causes the variables included in the “ALL” category in the panel data context.

This finding implies that past values of INS contain significant predictive information about the future values of the other variables in the model, supporting the presence of a dynamic causal relationship between INS and these variables. The robustness of this result, as evidenced by the highly significant *P* value, underscores the relevance of INS as a predictor in the panel VAR framework.

## DISCUSSION

This study aims to analyze how private sector hospitals in KSA, the UAE, and Jordan have responded to changes in INS. Empirical findings reveal predominantly negative associations, highlighting the need for a deeper exploration of system-level factors and health policy dynamics. Our results highlight the negative association between INS and the number of hospitals. This can be explained by the availability and quality of public healthcare, perceptions of healthcare accessibility, and the economic implications of increased hospital competition. These factors reduce the need for private HI. This aligns with the findings of Trish and Herring,^18^ Sepehri et al,^22^ and Wang et al^25^ that highlight the influence of social HI on hospital settings. Similarly, the relationship between INS and the number of hospital beds showed a significant negative association. This inverse relationship was elucidated by key factors such as the adequacy of public health care, where their healthcare needs are met without private HI, and in general the quality of health care is fairly acceptable for all patients whether insured or uninsured.^24^ For example, the availability of sufficient hospital beds in public facilities might ensure that patients can receive necessary care without needing to rely on private insurance.^16^ Conversely, insured people may have to pay out-of-pocket costs, as the private insurance company may not cover all hospital fees. This creates dependence on public hospitals, especially if there is a noticeable inadequacy of public healthcare resources, which directly affects the demand for additional insurance. Moreover, economic considerations like increased competition from expanded hospital infrastructure lower healthcare costs, leading individuals to prefer paying out-of-pocket instead of investing in private HI, further decreasing demand. Furthermore, we found a negative but insignificant association between DCR and HI, suggesting that insurance demand may not directly drive medical workforce growth, potentially due to the long lead time required to train new doctors and mismatches between policy focus and workforce planning. However, the studies of Wu et al^44^ and Dugan^20^ do not support this finding, as they mentioned that HI funding can enhance hospitals’ resources, such as raising doctors’ wages, purchasing advanced medical equipment, and providing training and professional development programs for doctors, thereby attracting more doctors. Additionally, HI funding can improve medical education, leading to more medical students and an overall increase in number of doctors.

A negative significant relationship between INS and the NRS was revealed by the study. This can be justified as insufficient investment in educating and training nurses leads to a shortage and increased competition for nurses, which will also increase prices and create more pressure on the hospital budget.^45^ In addition, higher healthcare costs and budget pressure may discourage hiring more nurses. Further, a report by the World Health Organization mentioned that bureaucratic delays (eg, laws and legislation),^46^ and a strong public healthcare system reduce the need for private HI as better public services lower the perceived benefits of private insurance.

The Granger causality test reinforces the SEM results’ validity as it proves a time-lagged, predictive relationship between future INS and healthcare infrastructure (HPS, BED, DCR, NRS). The findings indicate that the past values of the lag variables collectively offer meaningful predictive information for forecasting future values of the lead variable. This result contributes analytical nuance by demonstrating that insurance take-up is not just a cause but also an effect of systemic capacity, an argument not well-enough developed in previous research.

Practically, it suggests that the effectiveness of HI policies can rely on investments made in health infrastructure before or at the same time as insurance programs. To illustrate, those with increased hospital or staff capacity are likely to experience subsequent increases in insurance coverage because more services become accessible. In contrast, if insurance programs are introduced without simultaneous supply-side investments, bottlenecks, underutilization, and consumer dissatisfaction can ensue, affirming perverse feedback mechanisms that undermine policy success.

### Policy Implications

These findings carry key policy implications for HI program development and implementation in KSA, the UAE, and Jordan. First, the inverse relationship between INS and the number of private hospitals and hospital beds explains the significance of a well-developed and accessible public health system in determining insurance coverage. Policymakers should thus make sure that insurance reforms are linked to the current public healthcare infrastructure to prevent limited demand and second-best effects. Second, the study’s evidence of a 2-way relationship between HI and healthcare infrastructure implies that increasing insurance coverage needs to be supplemented with concurrent investments in hospital capacity and human capital. Rolling out insurance schemes without additional supply-side resources may lead to bottlenecks and patient discontent, which can reverse the success of these policy initiatives. The poor correlation between the demand for insurance and an increase in healthcare staff, especially nurses, calls for long-term measures aimed at education, training, and retention of healthcare professionals. Overcoming bureaucratic and legislative bottlenecks hindering the expansion of workforce and infrastructure is equally critical to facilitating the timely scaling of services.

Additionally, economic aspects like out-of-pocket payments and restricted insurance coverage reduce demand for private insurance, suggesting reforms geared toward improving affordability and comprehensiveness. Last, these results promote evidence-based, data-informed policymaking that takes the lagging interaction between insurance adoption and healthcare capacity into account and thus fosters more adaptive and better-performing HI policies. This is significant for important aspects of health systems performance. While insurance coverage is growing, infrastructure development is limited, which may impact allocative efficiency. Limited growth in the workforce, especially the nursing workforce, might raise cost-effectiveness and quality delivery issues. Therefore, policymakers should approach insurance reforms not just as financial instruments, but also as mechanisms for promoting efficient, equitable, and sustainable health systems.

### Limitations

This study is limited to private sector healthcare facilities in KSA, UAE, and Jordan. Hence, the findings may not be generalizable to other countries, and the public health sector context should be explained cautiously. Second, the smaller sample size of hospitals and healthcare facilities may reduce the statistical power and limits the ability to detect more nuanced effects or variations across different contexts. Third, the analysis employs methods based on panel data, including a Granger causality test to account for dynamic relationships in demand; it cannot allow for claims about causal mechanisms owing to omitted variable bias and unobserved confounding factors at the system level. Fourth, negative associations between demand for HI and healthcare infrastructure markers, including hospital beds and workforce metrics, may also be confounded by unmeasured factors, such as patients’ preferences, unique details of private insurance schemes, and regulatory contexts that are not specified. Fifth, compared with other industries, the long lead times and complicated nature of developing human resources in healthcare, especially for doctors and nurses, may not have fully captured the effects of changes in insurance demand on workforce development during the study timeframe, and may well have underrepresented the long-term impacts of insurance demand. Finally, data limitations related to the accuracy of insurance coverage, hospital resource allocation, and quality metrics could impact the robustness of the results. Utilizing larger data sets and the use of mixed-method approaches to explore much more complex interactions should be considered for further research.

## CONCLUSION

This study presents important insights into the intricate interplay between healthcare infrastructure and INS in the private hospital markets of KSA, the UAE, and Jordan. The results show largely negative correlations between insurance demand and major healthcare capacity measures like the number of hospitals, hospital beds, and nurses, which imply that an efficient public healthcare system lowers the dependency on private HI. Additionally, the dynamic interaction revealed by panel VAR Granger causality tests further emphasizes that insurance demand and healthcare infrastructure shape one another across time, highlighting the necessity for concerted policy action. Notably, increasing HI coverage without concomitant investments in healthcare capacity and the development of the workforce could result in inefficiencies and unserved demand. These findings highlight the need for comprehensive health system planning in concert with insurance reforms, infrastructure development, and workforce reinforcement to maximize the sustainability and effectiveness of HI plans in the region.

### Disclosures

The authors declare no competing interests.

## Supplementary Material

Appendix

STROBE Checklist

## References

[1] OECD Global insurance market trends 2023.

[2] Cigna healthcare All the different types of HI.

[3] CANa S., Koyuncu B., Işik A. (2020). Effects of some indicators on life expectancy at birth and infant mortality rates in Turkey. Revista Argentina de Clínica Psicológica.

[4] Shami E., Tabrizi J. S., Nosratnejad S. (2019). The effect of HI on the utilization of health services: a systematic review and meta-analysis. Galen Med J.

[5] Li L. (2011). The challenges of healthcare reforms in China. Public Health.

[6] Raghupathi V., Raghupathi W. (2020). Healthcare expenditure and economic performance: insights from the United States data. Front Public Health.

[7] Guth M., Garfield R., Rudowitz R. (2020). The effects of Medicaid expansion under the ACA: updated findings from a literature review. Kaiser Family Foundation.

[8] Owusu-Sekyere E., Chiaraah A. (2014). Demand for HI in Ghana: what factors influence enrollment?. Am J Public Health Res.

[9] Dror D. M., Majumdar A., Chakraborty A. (2018). The effect of consensus on demand for voluntary micro HI in rural India. Risk Manag Healthc Policy.

[10] Doiron D., Kettlewell N. (2020). Family formation and the demand for HI. Health Econ.

[11] Kolstad J. T., Kowalski A. E. (2012). The impact of health care reform on hospital and preventive care: evidence from Massachusetts. J Public Econ.

[12] Maleki R., Janatolmakan M., Fallahi M., Andayeshgar B., Khatony A. (2023). Intention to leave the profession and related factors in nurses: a cross-sectional study in Kermanshah, Iran. Nurs Open.

[13] Pilny A., Roesel F. (2020). Are doctors better health ministers?. Am J Health Econ.

[14] World Health Organization Community-based HI.

[15] World Health Organization Primary health care.

[16] Al-Hanawi M. K., Mwale M. L., Kamninga T. M. (2020). The effects of HI on health-seeking behaviour: evidence from the Kingdom of Saudi Arabia. Risk Manag Healthc Policy.

[17] Memon S., Afzal K., Memon A., Shaikh N., Manghrio U. (2022). The impact of HI on low birth-weight infants and mothers at a tertiary care hospital in Tabuk, Saudi Arabia: a retrospective case-control study. Cureus.

[18] Trish E. E., Herring B. J. (2015). How do health insurer market concentration and bargaining power with hospitals affect HI premiums?. J Health Econ.

[19] Dauda S. (2018). Hospital and HI markets concentration and inpatient hospital transaction prices in the U.S. health care market. Health Serv Res.

[20] Dugan J. (2020). Effects of HI on patient demand for physician services. Health Econ Rev.

[21] Hillebrecht M., Klonner S., Sauerborn R., Sie A., Souares A. (2021). The demand for HI in a poor economy: evidence from Burkina Faso. Econ Dev Cult Change.

[22] Sepehri A., Simpson W., Sarma S. (2006). The influence of HI on hospital admission and length of stay—the case of Vietnam. Soc Sci Med.

[23] Audibert M., Mathonnat J., Pelissier A., Huang X. X., Ma A. (2013). Health insurance reform and efficiency of township hospitals in rural China: an analysis from survey data. China Econ Rev.

[24] Abuosi A. A., Domfeh K. A., Abor J. Y., Nketiah-Amponsah E. (2016). Health insurance and quality of care: comparing perceptions of quality between insured and uninsured patients in Ghana’s hospitals. Int J Equity Health.

[25] Wang H., Zhang D., Hou Z., Yan F., Hou Z. (2018). Association between social HI and choice of hospitals among internal migrants in China: a national cross-sectional study. BMJ Open.

[26] Mukwena N. V., Manyisa Z. M. (2022). Factors influencing the preparedness for the implementation of the national HI scheme at a selected hospital in Gauteng Province, South Africa. BMC Health Serv Res.

[27] Central Bank of Saudi Arabia.

[28] Central Bank of UAE The Annual Report on the UAE Insurance Sector for 2021.

[29] Jordanian Ministry of Health Jordan Insurance Federation.

[30] Fornell C., Larcker D. F. (1981). Evaluating structural equation models with unobservable variables and measurement error. J Mark Res.

[31] Wang X., Zhen F., Huang X., Zhang M., Liu Z. (2013). Factors influencing the development potential of urban underground space: structural equation model approach. Tunn Undergr Space Technol.

[32] De Oña J., De Oña R., Eboli L., Mazzulla G. (2013). Perceived service quality in bus transit service: a structural equation approach. Transp Policy.

[33] Hair J. F., Black W. C., Babin B. J., Anderson R. E. (2010). Multivariate data analysis.

[34] Pesaran M. H. (2007). A simple panel unit root test in the presence of cross-section dependence. J Appl Econ.

[35] Pesaran M. H., Yamagata T. (2008). Testing slope homogeneity in large panels. J Econom.

[36] Westerlund J. (2005). New simple tests for panel cointegration. Econ Rev.

[37] Kao C. (1999). Spurious regression and residual-based tests for cointegration in panel data. J Econom.

[38] Sims C. A. (1980). Macroeconomics and reality. Econometrica.

[39] Abrigo M. R., Love I. (2016). Estimation of panel vector autoregression in Stata. Stata J.

[40] Berdiev A. N., Saunoris J. W. (2016). Financial development and the shadow economy: a panel VAR analysis. Econ Model.

[41] Akkaya M. (2021). FinEcon Handbook.

[42] Pesaran M. H. (2004). General diagnostic tests for cross-section dependence in panels. Cambridge Working Papers. Economics.

[43] Frees E. W. (1995). Assessing cross-sectional correlation in panel data. J Econom.

[44] Wu Y., Zhang L., Liu X., Ye T., Wang Y. (2018). Geographic variation in HI benefits in Qianjiang District, China: a cross-sectional study. Int J Equity Health.

[45] Loring S. Public school districts and the allocation of disaster aid: Evidence from Hurricane Harvey.

[46] World Health Organization Health workforce.

