# Supplementary material for: Private Sector Hospitals’ Response to Changes in Demand for Health Insurance in Arab Countries
Source: J Health Econ Outcomes Res. 2025 Jun 20;12(1):238–45. doi: 10.36469/001c.140416 (PMC12182724; doi:10.36469/001c.140416)
Supplement: Appendix [file jheor_2025_12_1_140416_288766.pdf]

Appendix

Variables	Definition	Websites
No. of hospitals (HPS)	Total number of private sector hospitals per 1000 population	Central Bank of UAE, Central Bank of Saudi Arabia, Ministry of Health, Jordan
No. of beds (BED)	Total number of beds in private hospitals per 1000 population	Central Bank of UAE, Central Bank of Saudi Arabia, Ministry of Health, Jordan
No. of doctors (DCR)	Total number of doctors working in private hospitals per 1000 population	Central Bank of UAE, Central Bank of Saudi Arabia, Ministry of Health, Jordan
No. of nurses (NRS)	Total number of nurses working in private hospitals per 1000 population	Central Bank of UAE, Central Bank of Saudi Arabia, Ministry of Health, Jordan
Demand for health insurance (HI)	Measured by gross written premiums, which is the total amount of insurance premiums received by an insurance company from policyholders	Central Bank of UAE, Central Bank of Saudi Arabia, Ministry of Health, Jordan
GDP per capita (GDPPC)	Gross domestic product divided by midyear population (constant 2015 USD).	World Development Indicators
Population growth (POP)	Annual population growth rate	World Development Indicators

Commented [AE1]: Consider hyperlinks for these websites.
